# Recent Advances in Understanding the Structure and Function of the Human Microbiome

**DOI:** 10.3389/fmicb.2022.825338

**Published:** 2022-02-03

**Authors:** Walaa K. Mousa, Fadia Chehadeh, Shannon Husband

**Affiliations:** ^1^College of Pharmacy, Al Ain University of Science and Technology, Al Ain, United Arab Emirates; ^2^Department of Biology, Whitman College, Walla Walla, WA, United States; ^3^College of Pharmacy, Mansoura University, Mansoura, Egypt

**Keywords:** human microbiome, disease associations, microbial diversity, probiotics, fecal transplant

## Abstract

Trillions of microbes live within our bodies in a deep symbiotic relationship. Microbial populations vary across body sites, driven by differences in the environment, immunological factors, and interactions between microbial species. Major advances in genome sequencing enable a better understanding of microbiome composition. However, most of the microbial taxa and species of the human microbiome are still unknown. Without revealing the identity of these microbes as a first step, we cannot appreciate their role in human health and diseases. A shift in the microbial balance, termed dysbiosis, is linked to a broad range of diseases from simple colitis and indigestion to cancer and dementia. The last decade has witnessed an explosion in microbiome research that led to a better understanding of the microbiome structure and function. This understanding leads to potential opportunities to develop next-generation microbiome-based drugs and diagnostic biomarkers. However, our understanding is limited given the highly personalized nature of the microbiome and its complex and multidirectional interactions with the host. In this review, we discuss: (1) our current knowledge of microbiome structure and factors that shape the microbial composition, (2) recent associations between microbiome dysbiosis and diseases, and (3) opportunities of new microbiome-based therapeutics. We analyze common themes, promises, gaps, and challenges of the microbiome research.

## Introduction

The human microbiome is the collection of microbes, and their associated genes and secreted molecules that live on or inside the human body (Gilbert et al., 2018). The microbial communities within our bodies are highly personalized and are considered as unique to each individual as their fingerprints (Franzosa et al., 2015) and are even unique to each body site (Dekaboruah et al., 2020). Many factors shape microbial diversity and abundance such as diet, host genetics, diseases, drugs, and lifestyle (Yadav and Chauhan, 2021a). The collection of these factors results in a microbial signature, that is, either a balanced and diverse healthy microbiota or an imbalanced and dysbiotic composition. Microbial dysbiosis is associated with the onset and progression of many diseases such as inflammatory bowel diseases (IBDs), obesity, metabolic disorders, and mental disorders (Gorecki et al., 2019; Wei et al., 2020; Doelman et al., 2021). While there is a plethora of research linking the microbiome dysbiosis to a particular disease, little is known about the underlying mechanisms (Liang et al., 2019; Gomaa, 2020; Duran-Pinedo et al., 2021; Lau et al., 2021). It is logical to anticipate that missing or enriched microbial taxa might affect microbial interactions and secreted metabolites which in turn can change host metabolism and other body functions. Various factors contribute to this microbiome-host interactions including ecological, epigenetics, and genetics components (Yadav et al., 2018). Understanding how microbial metabolites influence the health or disease status would have a significant impact on treating diet related diseases (Yadav et al., 2018). Few examples of microbial metabolites are known to contribute to the health and well-being of the body such as short-chain fatty acids (SCFAs; Segain et al., 2000), which contribute to intestinal homeostasis, reduce inflammation, and decrease gut permeability. Other microbial metabolites mediate diseases such as lipopolysaccharides which increase inflammation (Lin et al., 2020) and colibactin, which is implicated in colon cancer (Wilson et al., 2019). Understanding the mechanisms and secreted molecules underlying microbiome-disease associations will lead to innovative therapeutic interventions (Yadav and Chauhan, 2021b). Much interest exists in the therapeutic potential of the microbiome (Knight, 2010; Raes, 2014). There is an intriguing interest in the use of probiotic microbes or fecal transplantation to restore the balance of gut microbes. Preliminary data suggest that probiotics can treat diseases beyond the stomach disorders such as cancer and Alzheimer’s. Although, the approach of developing microbiome-based therapeutics or adjuvants is promising, it is far more complicated than anticipated. The main challenge is the lack of causality direction. For example, we do not know if a change in microbiota drives the disease or the disease itself modulates the microbiome. Another obstacle is the highly dynamic and personalized nature of the microbiome, which makes developing drugs that are universally usable very challenging. In this review, we discuss the current knowledge of microbiome structure, function, and future applications. The microbiome research is gaining tremendous interest as documented by the explosion in publications with more than 20,000 articles published in 2020 alone. The review combines and critically discusses recent advances in the field and how these advances contributed to better understanding of structure and function of the microbiome. This understanding will lead to future promises of the microbiome therapeutics. We conclude with future directions and how to convert the basic science into translational medicine and developing of innovative microbiome-based therapy.

## Understanding the Microbiome Composition and Factors That Shape Its Diversity

**Figure 1:**
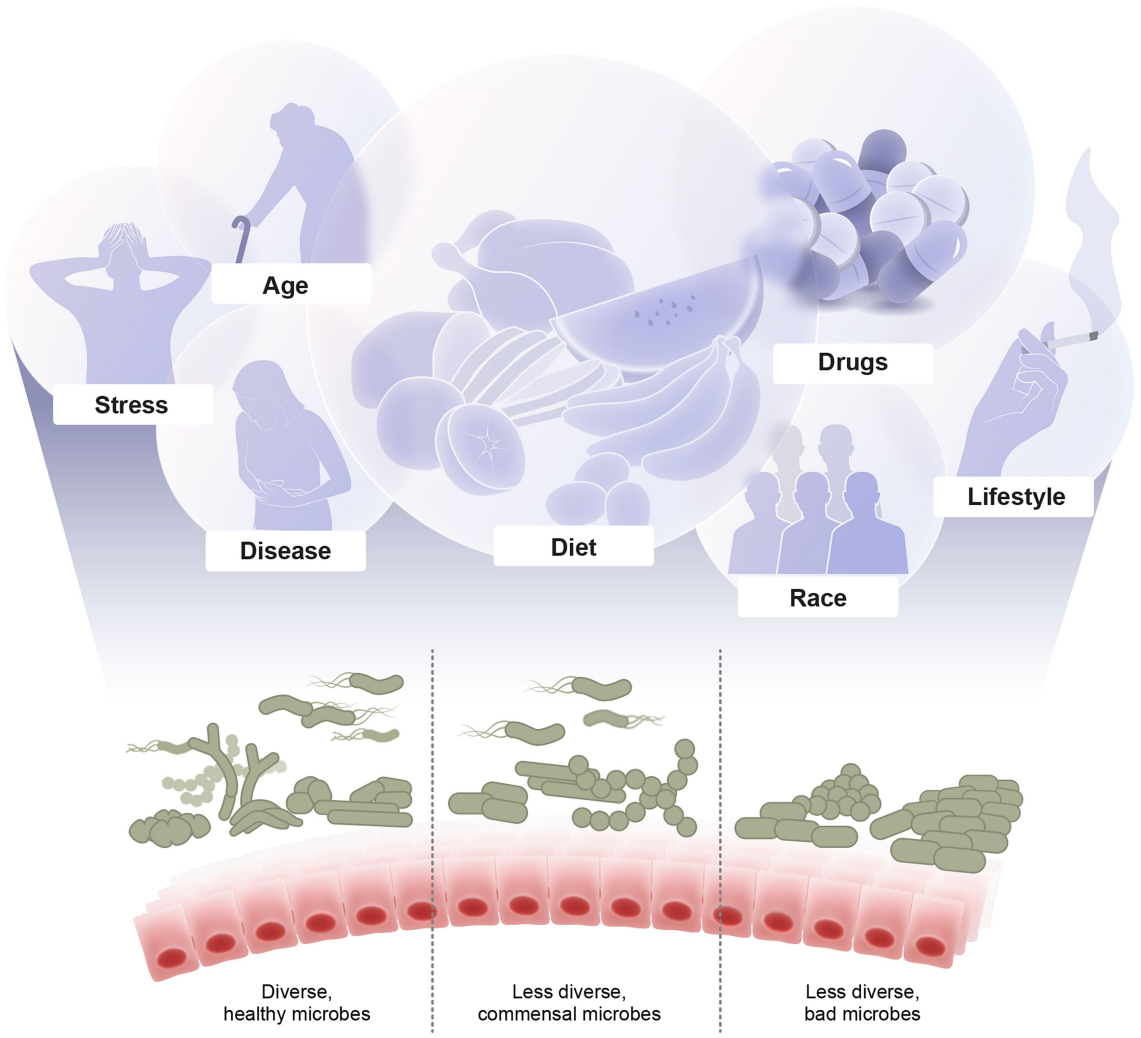
Major factors shaping the microbiome diversity. Illustrated are examples of multiple overlapping factors that shape the microbial composition including diet, stress, diseases, drugs, lifestyle, age, and host genetics. The outcome of these interacting factors are either healthy diverse, imbalanced, or dysbiotic microbiome which in turn affects the host health and diseases.

Multiple overlapping factors shape the microbiome composition. These factors include diet, stress, diseases, prescription and recreational drugs, smoking, drinking, aging, and others (Figure 1). Here, we discuss recent developments in revealing the implications of these factors and understanding the underlying mechanisms of how they modulate the microbiome.

### Effect of Diet Composition on the Microbiome Diversity

The interaction between diet, microbiome, and host is complex and multidirectional. Changing the diet regimen changes the microbiome composition. Different individuals’ microbiome responds differently to diet due to variations in food metabolism (Johnson et al., 2019). Similarly, different microbiome composition affects host metabolic capabilities. This bidirectional interaction further challenges our understanding of how the microbiome structure is shaped and the complexity of interacting factors from the external environment and host biology. Interestingly, changing the diet of immigrants from Asia to the United States is linked to an immediate and intense change in the microbiome structure with an impact on their health and development of obesity and its associated diseases (Vangay et al., 2018). The effect of diet on the onset and progression of certain diseases such as celiac disease and inflammatory bowel syndrome are now believed to be mediated by the microbiome and not a direct effect of the diet itself (Krishnareddy, 2019; Glassner et al., 2020). The beneficial effect of diet is also now thought to be mediated by the microbiome. For example, the health benefits of polyphenols are mediated through their promoting effect on *Akkermansia muciniphila* (Naito et al., 2018; Jayachandran et al., 2020). Similar links exist between dark chocolate and the microbiome structure. Dark chocolate is known for its anti-inflammatory properties due to its high content of antioxidant polyphenols (Magrone et al., 2017). A study showed that these antioxidants are poorly absorbed but could be fermented by colon bacteria such as *Bifidobacterium* into smaller polyphenolic polymers with anti-inflammatory properties that decrease cardiovascular inflammation (Wiese et al., 2019).

#### Macronutrients and Microbiome Diversity

Evidence is mounting for the effect of diet on modulating the microbiome diversity, which in turn modulates the immune function and metabolic reactions. Fermented food such as kimchi, kombucha tea, and kefir shows positive enhancement of microbiome diversity associated with a significant decrease in inflammation biomarkers. Interestingly the affected biomarkers such as interleukin 6 are implicated in other diseases beyond the gastrointestinal tract (GIT) such as chronic stress, rheumatoid arthritis, and diabetes type 2 (Lau et al., 2021; Wastyk et al., 2021). This effect is observed in all participants of the cohort after only 10 weeks of incorporating fermented food into their diet. The uniqueness of this study is suggesting that only one change in the diet, and for a very short time, leads to a significant positive change in microbiome structure without the need to remodel the entire diet regime. An outstanding question regarding the mechanistic underpinning of the variation in response to different food remains.

In contrast to fermented food, the high fiber diet does not change the microbiome structure at the same time frame (Lau et al., 2021; Wastyk et al., 2021). One possible mechanism might be the lack of fiber degrading microbes. One of the well-known roles of gut microbiota is their ability to ferment indigestible dietary fibers to produce SCFAs. Another study shows that the incorporation of dietary fibers into the diet for 2 weeks only changes the microbiome composition in 20% of the studied subjects (Johnson et al., 2021; Lau et al., 2021). This observed effect is dependent on the original microbiome composition and the amount of fiber consumed (Johnson et al., 2021; Lau et al., 2021). The responder’s microbiome is enriched in *Prevotella copri* and shows less abundance of Bacteroides. These studies give us a new insight into the bidirectional interaction between dietary fiber and the microbiome. Curiously, we question whether adding probiotics will enable the existing microbiome to better benefit from dietary fibers or combining different food items can modulate microbiome response. In post-menopausal women, a very low-calorie diet, consisting of oats, greek yogurt, or fish, is associated with increases in *Akkermansia* sp., known for its ability to metabolize host glycan, and decreases in genera such as *Roseburia*, *Ruminococcus*, and *Eubacterium*. This microbiome shift is associated with high levels of SCFAs. Fecal transplant of this altered microbiome to germ-free (GF) mice resulted in the overabundance of the pathogen *Clostridium difficile* (von Schwartzenberg et al., 2021).

#### Nutrient and Mineral Supplements and Microbiome Diversity

Similar to diet, nutrient supplements such as vitamins and minerals affect the microbiome composition (Merlen, 1986; Zimmer et al., 2012). In mice, low iron intake increases specific taxa such as Lachnospiraceae, Ruminococcaceae, and Rikenellaceae. These taxa are thought to regulate gut transport pathways to retain iron and decrease its excretion to maintain the iron level in the blood (Coe et al., 2021; Lau et al., 2021). Interestingly, *Lactobacillus* can sense iron deprivation and communicate the iron need to a murine host *via* the production of small-molecule mediators that interact with a hypoxia-inducible factor, HIF-2a (Das et al., 2020). Among these small molecules, reuterin and 1,3-diaminopropane (DAP) are shown to suppress intestinal HIF-2a activity both *in vitro* and *in vivo*. Specifically, reuterin and DAP prevent HIF-2a dimerization with aryl hydrocarbon receptor nuclear translocator (ARNT). It is interesting to speculate that iron deficiency selectively favors the growth of microbes and metabolites that exert high levels of HIF-2a inhibition. Microbial metabolites are known to affect the stability of HIF-2a, which in turns affects the intestinal hemostasias and gut barrier functions as comprehensively reviewed (Kumar et al., 2020). The microbiome can promote cellular iron storage *via* the induction of an iron storage protein, FTN, which also increases iron absorption (Vanoaica et al., 2010). It is still unclear though how microbes and their host compete during times of iron scarcity. Other studies show that high Na^+^ intake linked to hypertension alters gut microbial composition in mice increasing the relative abundance of several bacterial taxa such as *Prevotella* and Bacteroides. Prevotellaceae is linked to metabolic syndrome and chronic inflammation (Barbaro et al., 2017; Ferguson et al., 2019; Van Beusecum et al., 2019). High salt intake stimulates the production of pro-inflammatory markers such as IL-17, and, coupled with a defect in the regulation of natriuresis, which is associated with hypertension. Transplanting gut microbiomes from hypertensive subjects increases blood pressure in GF recipient mice, suggesting a causal role of the gut microbiome in the development of Na^+^ induced hypertension (Elijovich et al., 2020).

### Stress

Stress remodels the gut microbiota and hence affects disease susceptibility (Kelly et al., 2015; Zijlmans et al., 2015). Studies show that social stress exposure decreases the abundance of microbes with anti-inflammatory activity such as para Bacteroides taxa (Maltz et al., 2018, 2019), which in turn decreases microbial anti-inflammatory metabolites such as SCFAs and contribute to a higher level of inflammation. Combined stress and infection or other inflammatory diseases worsen the outcome of the disease compared to non-stressed subjects. Consuming microbes known for anti-inflammatory activity might be beneficial for people with anxiety disorder and unmanageable stress levels.

Some studies found that depressed patients possess a higher level of *Bacteroidales* and a lower level of *Lachnospiraceae* (Naseribafrouei et al., 2014). Another study shows increased levels of *Enterobacteriaceae* and *Alistipes* in depressed patients (Jiang et al., 2015a). High stress during pregnancy is also associated with a change in vaginal *Lactobacillus* (Jašarević et al., 2015a). The consequences are also extended to the newborn babies from high-stress mothers, who show a higher abundance of *Proteobacteria* and lower abundances of *Lactobacillus* and *Bifidobacteria* (Zijlmans et al., 2015). This altered microbiome in infants results in a series of pathological conditions such as gut inflammation and the development of allergies (Zijlmans et al., 2015). Mouse offspring exposed to maternal stress had decreased levels of *Bifidobacteria* and Lactobacilli, associated with decreased cognitive functioning and increased anxiety (Golubeva et al., 2015; Jašarević et al., 2015b). Exposure to stressors can significantly change the microbial populations in the gastrointestinal tract of both humans and laboratory animals. These microbiota changes are more pronounced several days after exposure to a stressor than on the day of or the day after the exposure. In mice, exposure to stress results in microbiota shifts that are characterized by a lower abundance of Porphyromonadaceae, and increases in aerobic bacteria (Bailey et al., 2010). Stressed mice exhibited greater susceptibility to infections with the pathogen *Citrobacter rodentium*. Stressed mice show higher levels of tumor necrosis factor (TNF) and suppression of the anti-inflammatory cytokine IL-10 before the pathogen challenge, suggesting an immunomodulatory effect of dysbiosis microbiota, which renders the host more susceptible to infection (Bailey et al., 2010). Stress-induced inflammation affects the integrity of the tight junction resulting in leakage of molecules through gastrointestinal barriers and translocation of gut bacteria to the liver and spleen (Moussaoui et al., 2014). Probiotic strains such as *Lactobacillus helveticus* R0052, *Bifidobacterium longum* R0175 (Ait-Belgnaoui et al., 2012), and *Lactobacillus farciminis* (Ait-Belgnaoui et al., 2012) show promise in the treatment of stress-related disorders in mice.

### Drugs

It is commonly accepted that antibiotics, in a particularly broad-spectrum, alter the composition of gut microbiota which can last for months and even a year (Looft and Allen, 2012; Konstantinidis et al., 2020). Research shows that other non-antibiotics can also affect the composition and diversity of gut microbiota in population-based cohorts (Nichols et al., 2019; Papazafiropoulou, 2019). Drugs such as oral steroids, platelet aggregation inhibitors, antidepressants, and vitamin D supplements increase the abundance of *Streptococcus salivarius* (Vila et al., 2020; Weersma et al., 2020). Benzodiazepine was found to be associated with an increase in the abundance of *Haemophilus parainfluenzae*, a bacterium that has been reported to be more common in patients with Irritable Bowel Syndrome (Vila et al., 2020). Proton pump inhibitors selectively increase the abundances of *Bifidobacterium dentium salivarius* (Vila et al., 2020). SSRI antidepressants increase the abundance of *Eubacterium ramulus*, while tricyclic antidepressant increase the abundances of Clostridium *leptum* (Vila et al., 2020). Laxatives enrich *Alistipes* and Bacteroides species (Vila et al., 2020; Weersma et al., 2020). Steroids increase the abundance of *Streptococcus mutans* and *B. dentium*.

Addictive substances implicated in substance use disorder (SUD), such as opiates, cocaine, psychostimulants, and alcohol, also modulate the microbiome (Angoa-Pérez and Kuhn, 2021). Almost all research on SUD has primarily focused on the underlying CNS elements and ignored the microbiome link. Given that SUD affects millions every year, it is of intriguing importance to shed a light on the modulatory effect of abuse drugs on the gut microbiome. This research will help to understand subsequent consequences on the health and diseases of the host and to develop better therapeutics to tackle the SUD crisis. Some nutrients such as sugar and fats can exert an addictive effect like drugs of abuse. The similarity includes the development of dependence and withdrawal behavior and induction of dopamine release (Volkow et al., 2011). However, it is not known yet if the effect of diet on dopamine and brain reward mechanism is mediated by a change in the microbiome composition, which affects the gut-brain axis. Data collected from studying the effect of alcohol consumption on experimental animals show that alcohol can alter gut microbiota composition not only in high chronic doses but also even with mild and moderate consumption (Barr et al., 2018). Similar to stress, alcohol consumption can also increase intestinal permeability, alter the microbiome, and increase depression, these conditions are improved after withdrawal (Kelly et al., 2015).

### Race and Host Genetics

Host genetics are the least understood factor in shaping the microbiota (Gaulke and Sharpton, 2018). Some studies show that genetic mutation results in atypical microbiota collection in mice (Buffington et al., 2021; Lau et al., 2021). A comparative analysis on 73 Argentinian participants living in United States and United Kingdom showed that the microbiome composition of obese patients showed a lower abundance of Porphyromonadaceae, Rikenellaceae, Bacteroidaceae, Tenericutes, and Verrucomicrobia. Other taxa such as *Roseburia*, *Ruminococcus*, *Blautia producta*, *Eubacterium biforme*, *Clostridium lactatifermentans*, and *Ruminiclostridium* showed higher abundance. While *Lactobacillus* remains unaltered. Interestingly, the gut microbiota of normal weight, overweight, and obese subjects clustered separately based on geography revealing a significant difference in their microbiota composition. For example, Ruminococcaceae taxa dominate samples collected in the United Kingdom, while Lachnospiraceae taxa are the most abundant taxa in samples collected from the United States. This microbial clustering results in a variation of the host metabolism and risk of developing diseases such as obesity (Lau et al., 2021; Pesoa et al., 2021). Residents of low-income countries show higher diversity in their microbiome compared to West countries. Interestingly, data show that immigrants from Southeast Asia to the United States lose 15% of their microbiome diversity immediately (Vangay et al., 2018). This loss of microbiome diversity is associated with the incidence of some diseases such as obesity and cardiovascular disorders. The loss of diversity might be due to a combination of factors such as the shift to a western diet with high calories, the drinking water, or the use of drugs and antibiotics (Vangay et al., 2018).

### Aging

Understanding the development of microbiome profiles over time, as individuals age, remains an elusive challenge for researchers. In older populations especially, studies show associations between gut microbiome composition and physical fitness, diet, frailty, and mental capacity demonstrating the importance of a robust functioning microbiome (Lau et al., 2021; Wilmanski et al., 2021). Changes in the microbiome structure of older individuals have often been attributed to altered lifestyles, diets, reduced mobility, decreased immune function, reduced intestinal capability, changed gut morphology, increased use of medication and drugs, and recurrent infections (Kim and Benayoun, 2020). There remains some obscuring around the directions of causality in microbiome-aging interactions: are changes in gut microbial composition caused by reduced capability in older individuals, or are gut microbes the driving force behind symptoms of aging? The relationship between microbiome composition and aging is likely bi-directional, despite relationships of causality remaining ambiguous. However, patterns of association in the gut microbiomes of older individuals have been elucidated. Within three independent cohorts, comprising 9,000 individuals, researchers identified a pattern of healthy aging characterized by a depletion of core gut microbial taxa, namely *Bacteroides*, with healthier individuals having increasingly distinct microbiome compositions compared to other members within the study (Lau et al., 2021; Wilmanski et al., 2021). Another study, examining the microbiome compositions of “semi-supercentenarians,” individuals aged 105–109 years, found that aging is marked by increasing abundance of subdominant species, with decreases in dominant core microbiota within the Ruminococcaceae, Lachnospiraceae, and Bacteroidaceae families (Biagi et al., 2016). Semi-supercentenarians showed peculiarities in their microbiome compositions, indicating enrichment of health-associated bacterial groups such as *Akkermansia* and *Bifidobacterium*, which contributed to the maintenance of good health during aging. Another study examined the differences in the microbial composition of young adults, individuals in their seventies, and centenarians (Biagi et al., 2010). Results show that the microbiome composition of young adult and 70-year-old microbiomes are highly similar, while the centenarians experienced decreases in abundance of *Faecalibacterium prausnitzii*, with 10-fold increases in *Eubacterium limosum*. These shifts in microbial populations in centenarians are associated with increased inflammation. The social activity of elder peoples plays a role in shaping their microbiome composition. Elderly individuals living in long-term facilities showed a higher abundance of *Bacteroidetes*, *Parabacteroides*, *Eubacterium*, *Anaerotruncus*, *Lactonifactor*, and *Coprobacillus*. The elderly who has greater exposure to the outside communities showed a higher abundance of *Firmicutes* and Lachnospiraceae, which are associated with higher levels of SCFAs. High fiber diets are associated with more diverse microbiomes. One can postulate a link between the shift in microbial composition to age-related health deterioration. If proven true, this will open a very interesting avenue for microbiome-based therapeutics to slow down aging and related diseases (Claesson et al., 2012).

### Lifestyle

Lifestyle has a profound impact on microbiome composition and diversity. Lifestyle is a broad term that might include physical activity, smoking, drug abuse, and the surrounding environment. The level of physical activity, as well as the amount of exposure to pollutants, are all critical factors affecting microbiome diversity.

#### Exercise and Microbiome Composition

Exercise increases microbiome diversity (Dalton et al., 2019) and subsequently modulates the gut-brain access (Kim et al., 2018; Dalton et al., 2019). The microbiome of physically active individuals is more diverse with greater abundances of health-promoting bacteria including *F. prausnitzii*, *Roseburia hominis*, and *A. muciniphila* (Bressa et al., 2017). In young adults, a relationship between cardiorespiratory fitness and microbiota composition is also evident from the increased abundances of *Firmicutes* in fit young adults (Durk et al., 2019). A study wherein lean and obese individuals participated in 6 weeks of exercise training revealed that exercise induced a significant microbiome shift. This shift is characterized by higher abundances of SCFAs-producing bacteria such as *Faecalibacterium* and *Lachnospiraceae* and an increase in SCFAs in feces of non-obese participants (Allen et al., 2018).

#### Smoking and Microbiome Composition

Cigarette smoke is a complex mixture of more than 7,000 chemicals depositing directly to the lung and gut. These chemicals affect the microbiome composition increasing susceptibility to various diseases such as infections and inflammatory disorders (Rogers et al., 2012; Lee et al., 2018). Even E-cigarette significantly alter the oral microbiome with the increase in *Veillonella* and *Haemophilus* (Chopyk et al., 2021; Lau et al., 2021). Interestingly, the nose of smokers shows a higher colonization rate with *Staphylococcus aureus* in E-cigarette smokers (Chopyk et al., 2021; Lau et al., 2021). The gut microbiome of smokers is enriched in *Bacteroides* and depleted in *Firmicutes* and *Proteobacteria* when compared to non-smokers and past smokers (Lee et al., 2018). The microbiome of the small intestines shows a higher abundance of *Firmicutes* such as *Veillonella* and *Streptococcus* and a lower abundance of *Prevotella* and Neisseria. The abundance of *Firmicutes* is partially restored after stopping smoking (Shanahan et al., 2018). The oral microbiome is impacted by cigarette smoking with lower abundances of *Proteobacteria*, *Capnocytophaga*, *Peptostreptococcus*, and *Leptotrichia* and enrichment of *Atopobium* and *Streptococcus* (Wu et al., 2016). Depletions of bacterial taxa were associated with functional shifts in smokers’ oral microbiomes mainly the functions related to carbohydrate, energy expenditure, and xenobiotic metabolism. A study in mice shows that the change in microbiome extends to thirdhand smokers with a persistent effect on metabolism (He et al., 2021; Lau et al., 2021).

#### Urbanization and Microbiome Composition

The environment alters the microbiome composition and subsequently mediates susceptibility to diseases (Littleford-Colquhoun et al., 2019). Westernization is linked to the decrease in microbiome diversity and increased incidence of diseases such as obesity and infectious diseases (Winglee et al., 2017). The difference in the environments includes multiple factors such as dietary culture, income, pollutions, and lifestyle. All of these variants change the microbiome. Young adults residing in Southern California, who are more exposed to atmospheric ozone, show lower gut microbial diversity with increased abundances of *Bacteroides caecimuris* (Fouladi et al., 2020). An interesting study profiled the microbiome from remains of ancient human feces and found a similarity to pre-industrial humans. Ancient microbiome shows higher abundances of *Enterococcus*, *Treponema succinifaciens*, and *Ruminococcus champenellensis*. These species might be linked to the consumption of complex carbohydrates (Lau et al., 2021; Wibowo et al., 2021). Humans in industrialized societies show microbiome enriched in *A. muciniphila*, *Alisipes*, and *Bacteroides* species. Industrialization is seemingly linked to higher abundances of antibiotic resistance genes compared to the microbiome of the pre-antibiotic era. A higher rate of horizontal gene transfer is also linked to the industrialization era, which might facilitate acquiring of new functions and adaptation to the change in the environment (Groussin et al., 2021; Lau et al., 2021). A pioneering study shows that the skin microbiome is altered in the urban population and this alteration changes the physiological response of the skin and is implicated in skin-related diseases (Lau et al., 2021; Wang et al., 2021c).

## Understanding the Microbiome Function and Its Association With Onset and Progression of Many Diseases

**Figure 2:**
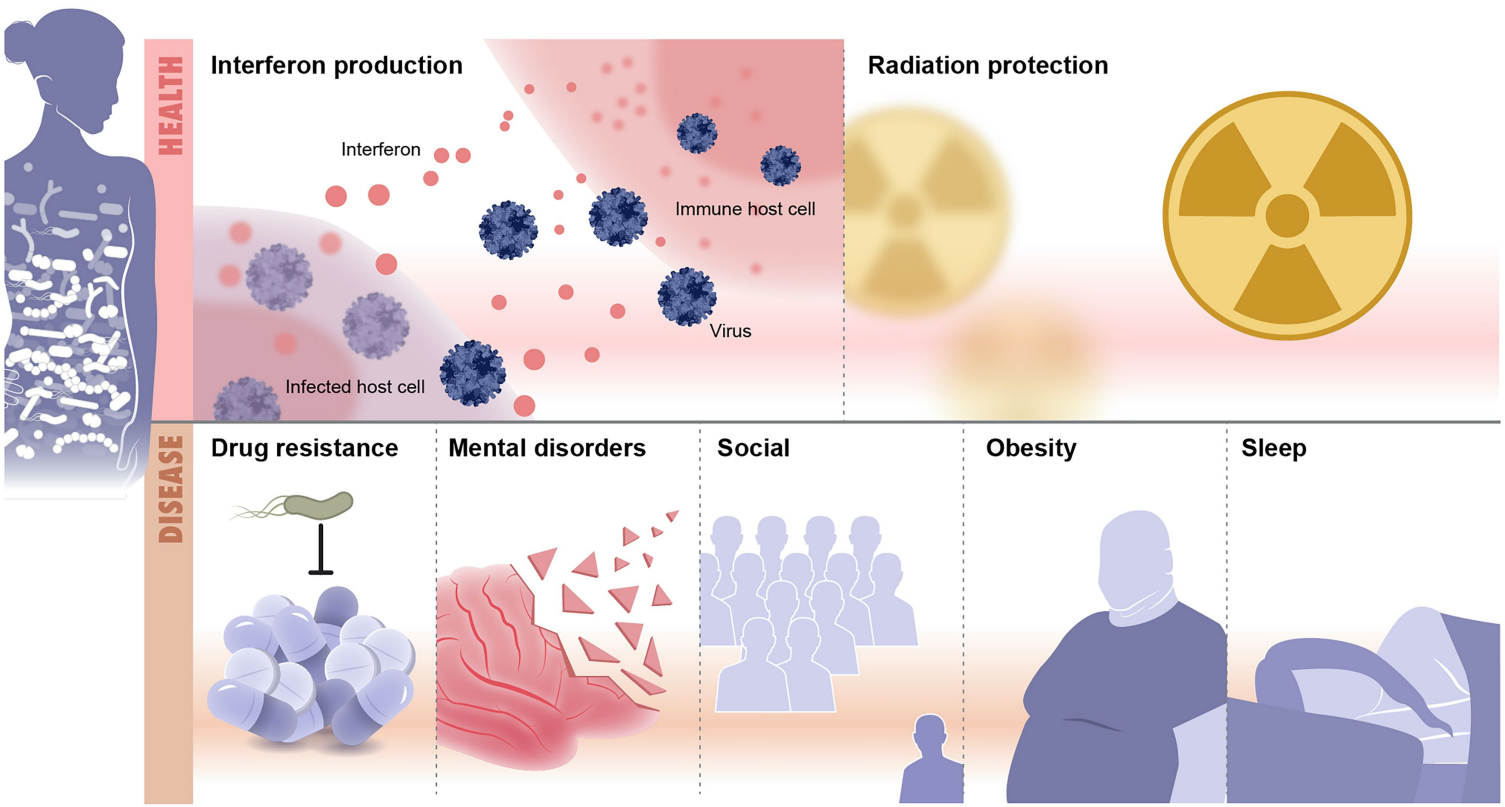
Microbiome and diseases association. Illustrated are examples of health and disease conditions that are linked to change in the microbiome. Health conditions associated with healthy microbiome include, as examples, protection from infections and radiation resistance. Diseases that result from imbalanced microbiomes are countless and include drug resistance, mental disorders, social diseases, obesity and metabolic syndrome, and sleep disorders.

Recent advances in microbiome research deepen our understanding of microbiome function either in mediating or preventing diseases. Although lacking details of underpinning mechanisms, these studies still provide valuable insights on the depth and altitude of microbiome impact on our health and diseases. Methods used to analyze the microbiome are summarized (Table 1). Here, we discuss recent microbiome associations with diseases from inflammation to neurological and mental illness (Table 2; Figure 2).

**Table 1:** Methods for the microbiome analysis.

Method	Description	Advantages	Limitations	References
Germ free (GF) models	Transplant *in vitro* embryos into germ-free mothers and raise animals without microorganism contact	“Blank slate” system used to test previously observed associations	Compromised, expensive systems which are not representative of natural microbiome functioning	Guo et al., 2020; Sani et al., 2021
Human sampling	Population is divided into subgroups based on specific characteristics	Cost effective and relatively easy to access body sample sites	Distinctions in regional composition are difficult to capture	Berg et al., 2020; Wang et al., 2021b
Population scale	Involves sampling from a selected large group of individuals	Large-scale conclusions can be drawn, with broadly applicable results	Diversity within individual microbiomes is not considered, with purely association-based results	Goyal et al., 2021
*In vitro* modeling	Experimental laboratory systems mimicking processes occurring within a living organism	Enables examination of relationships between specific microbes and host	Systems lack host-level complexity due to reduced microbial communities and simplified environmental structuring	Das et al., 2020
Co-occurrence network patterning	Explore interplay between organisms and environmental conditions on community interactions	Relationships between microbes and host-microbe interactions can be studied to determine ecological network components within microbiomes	Microbial community complexity is reduced, with subsequently simplified system functioning	Barbaro et al., 2017
Direct observation *via* fluorescence	Probe specific sites or organismal components such as cells, allowing microscopic observation	Taxonomy, locality and community organization can be evaluated and screens for specific phenotypes are possible	Photobleaching can occur	Wibowo et al., 2021
Bioinformatics	Use of software tools to understand biological data, especially with large, complicated data sets	Allows for rapid organization and analysis of data	Often expensive, while drawing association-based conclusions	Faust et al., 2012; Barbaro et al., 2017
Association studies	Identify genes correlated with disorders	Can discover correlative relationships between microbes and their hosts	The mechanisms and causative factors underlying correlations remain unknown	Carlson et al., 2021; Jin et al., 2021
Meta-omics	Includes metagenomic, metatranscriptomic, metaproteomic, and metabolomic data collection	Analyze and detect molecular and genetic components and mediators and metabolic profiles	Equipment is highly sensitive and expensive, limiting reproducibility	He et al., 2018a
Predictive machine learning models	Use of algorithms to identify patterns and behaviors within datasets	Employ the simplicity of *in situ* analysis to detect links between microbes and covariates	Difficulty in capturing the complexity of individual microbiomes, with association-based and time consuming data acquisition	Maifeld et al., 2021

**Table 2:** Microbiome shift and diseases.

Disease	Main microbiota shift	Subjects	Design	References
Hypertension	Increase in the abundance of *Prevotella*, *Bacteroides*, and *Faecalibacterium*	Smokers with hypertension (S-HTN), nonsmokers with HTN (NS-HTN), and smokers without HTN (S-CTR)	Fecal sample analysis and metagenomic sequencing	Wang et al., 2021a
	Increase in the abundance of opportunistic pathogens such as *Klebsiella* spp., *Streptococcus* spp., and *Parabacteroides merdae*	Human subjects	Metagenomic sequencing	Yan et al., 2017
Pancreatic ductal adenocarcinoma (PDA)	Reduction in *Firmicutes* and increase in the phylum *Proteobacteria* in patients with PDAC	Human subjects	Metagenomic sequencing	Zhou et al., 2021
	Abundance of *Klebsiella pneumoniae*, *Clostridium bolteae*, *Streptococcus mutans*, and two *Parabacteroides* spp.	Human subjects	Whole genome sequencing	Matsukawa et al., 2021
Fear	A decrease abundance of *Bacteroides* and increase in *Veillonella*, *Dialister*, *Bifidobacterium*, *Lactobacillus*, and *Clostridiales*	Infants	Association study	Carlson et al., 2021
	Increase in *Clostridia* spp., and *Enterobacter* spp.	Experimental animals (mice)	Metagenomic analysis	Chu et al., 2019
Coronary artery disease (CAD)	CAD: increase in the order *Lactobacillales*, and a decrease in the phylum *Bacteroidetes*	Human subjects	Review	Yamashita et al., 2016
	CAD: reduction in the abundance of *Roseburia Intestinalis* and *Faecalibacterium prausnitzii*	Human subjects	Metagenome-wide association	Jie et al., 2017
Lung disease	Increase in the abundance of *Legionellales*	Leaves and soil	Culture-based sampling methods, sequencing, and cloning	Solbach et al., 2021
	Downregulation in *Alloprevotella*, *Helicobacter*, and *Rikernella*. Upregulation in *Dubosiella*, *OIsenella*, and *Parasutterella*	Experimental animals (mice models)	Metagenomic analysis	Gong et al., 2021
Neurodegenerative disease	Increase in the abundance of *K. pneumoniae* and *Pseudomonas aeruginosa*	Experimental animals (*C. elegans*)	Culture-based method and metagenomics	Walker et al., 2021
	Decrease in *Blautia*, *Coprococcus*, and *Roseburia*			Zhu et al., 2020
Severe mental disorder (SMD)	A decrease in the abundance of *Bifidobacteria* and *Lactobacilli*	Experimental animals (GF mice)	Meta analysis	Sani et al., 2021
	Increase in the abundance of *Clostridiales*, *Lactobacillales*, and *Bacteroidales*	Human subjects: high risk (HR) and ultra high risk (UHR) participants	Metagenomic analysis	He et al., 2018b
Anxiety	Increase in *Ruminococcaceae* and *Clostridiales*	Experimental animals (mice)	Genome-wide associations study	Jin et al., 2021
	Decrease in *Bacteroidaceae*, *Clostridiaceae*, and *Bacteroidales*		Metagenomic analysis	Buffington et al., 2016
Depression (MDD)	Increase in the abundance of *Alistipes*, *Flavonifractor*, *Butyricimonas*, *Clostridium XlVb*, *Phascolarctobacterium*, *Bacteroidetes*, and *Proteobacteria*	Human subjects	Fecal sample analysis	Jiang et al., 2015
	A decrease in the abundance of *Faecalibacterium*			Yang et al., 2021
	Increase in *Bacteroides* and a decrease in *Blautia* and *Eubacterium*	Human subjects	Metagenomic analysis	
Alzheimer’s disease: mild cognitive impairment (MCI)	A decrease in the proportion of *Ascomycota*, *Cladosporiaceae*, and *Meyerozyma*	Human subjects	Fecal sample analysis and metagenomic analysis	Nagpal et al., 2020
	Increase in the proportion of *Basidiomycota*, *Sclerotiniaeae*, *Phaffomyceteceae*, *Trichocomaceae*, Cystofilobasidiaceae, Togniniaceae and genera Botrytis, Kazachstania, Phaeoacremonium, and Cladosporium			
	Increase in the relative abundance of *Fusobacterium*, *Bacteroides*, *Prevotella*, *Peptoclostridium*, and *Alloprevotella*	Experimental animals (dogs)	Metagenomic analysis	Kubinyi et al., 2020
	A decrease in the proportion of *Actinobacteria*			
Dementia and bipolar disorder	Increase in the frequency of *Aggregatibacter actinomycetemcomitans* and *Porphyromonas gingivalis*	Human subjects	Meta analysis	Maitre et al., 2020
	Dementia group: Lower number of *Bacteroides*. Frequent *Lactobacillales* and *Bifidobacterium*	Human subjects	Metagenomic analysis	Saji et al., 2019
COVID-19	Increase in the abundance of opportunistic microbes such as *Candida albicans* and *Clostridium*	Human subjects	Literature review	de Oliveira et al., 2021
	Increase in the abundance of *Bacteroidetes*, *Actinobacteria*, *Ruminococcus gnavus*, *Ruminococcus torques*, and *Bacteroides dorei*	Human subjects	Fecal sample analysis and metagenomic sequencing	Yeoh et al., 2021
	A decrease in the abundance of *Bifidobacterium adolescentis*, *Faecalibacterium prausnitzii*, and *Eubacterium rectale*			
Autism spectrum disorder	ASD: decrease in *Faecalibacterium*, *Bifidobacteria*	Human subjects	Meta analysis	Zhu et al., 2020
	CVD: increase in *Lactobacillus Enterobacter*, and *Bacteroides*			Ye et al., 2021
	ASD: decrease in the relative abundance of *Escherichia*, *Shigella*, *Veillonella*, *Akkermansia*, *Provindencia*, *Dialister*, *Bifidobacterium*, *Streptococcus*, *Ruminococcaceae UCG_002*, *Megasphaera*, *Eubacterium_coprostanol*, *Citrobacter*, *Ruminiclostridium_5*, and *Ruminiclostridium_6* and increase in *Eisenbergiella*, *Klebsiella*, *Faecalibacterium*, and *Blautia*	Human subjects	16S ribosomal RNA gene sequencing and metagenomic sequencing.	
Sleep	Mid sleep fragmentation (SF) had lower *Firmicutes*:Bacteroides ratio and a decreased alpha diversity	Experimental animals (rats)	Fecal sample analysis and metagenomic analysis	Maki et al., 2020
	Greater abundance of *Proteobacteria* in SF rats			
	Lower abundance of *Sutterella* and higher abundance of *Pseudomonas*	Humans subjects	Metagenomic analysis	Agrawal et al., 2021
Obesity	Increase in the abundance of *Firmicutes* and lower Bacteroidetes proportion in obese individuals	Human subjects and animal models	Literature review	Tseng and Chun-Ying, 2019
	A decrease in the abundance of several Bacteroidetes taxa such as Flavobacteriaceae, Prphyromonadaceae, and Sphingobacteriaceae	Human subjects	Metagenomic analysis	Palmas et al., 2021

### Microbiome Association With Inflammatory and Metabolic Disorders

#### Chronic Inflammation in GIT and Beyond

The gut microbiome mediates mucosal immune response and directly implicates the inflammation process. Some microbiome species are known to produce SCFAs that decrease inflammation. Others are implicated in causing GIT inflammation that might progress into chronic conditions. Several review articles discussed the role of the microbiome in GIT inflammation and immune response (Abdulla et al., 2021; Sanders et al., 2021). Beyond GIT, inflammation is linked to intrauterine growth restriction (IUGR) in humans (Al-Azemi et al., 2017). However, there is no data available on the interplay between gut microbiota, proinflammatory cytokines, and IUGR (Fernandez-Gonzalez et al., 2020). Some data suggest that the administration of *Lactobacillus* strains improves glucose metabolism and decreases the risk of preeclampsia in pregnant women (Myhre et al., 2011). In livestock, high dietary fiber intake shows a significant decrease in inflammation and IUGR in pregnant animals (Liu et al., 2021). Identifying microbial strains that can improve pregnancy outcomes such as IUGR, pregnancy diabetes, preeclampsia, and other pregnancy complications is an important area of research that will inform advanced care regimens including the development of probiotics. Chronic inflammation is linked to the development of some cancers of the colon and pancreas.

#### Microbiome Implications in the Development of Malignant Tumors

Recent research demonstrates a link between dysbiosis in gut microbiota and cancer development (Ammer-Herrmenau et al., 2020; Lau et al., 2021; Matsukawa et al., 2021). Some microbes are more abundant in Pancreatic Ductal Adeno Carcinoma (PDAC) patients including *Klebsiella pneumoniae*, *Clostridium bolteae*, *S. mutans*, and *Parabacteroides* spp., *Acinetobacter*, and *Pseudomonas* (Thomas and Jobin, 2020; Lau et al., 2021; Matsukawa et al., 2021). The abundance of other microbes such as *Firmicutes* is significantly lower in PDAC patients (Lau et al., 2021; Zhou et al., 2021). Most PDAC patients suffer from a leaky gut which results in the translocation of harmful bacteria into the bloodstream (Wu et al., 2014). These bacteria can trigger an immune response through pathways that involve tumor-infiltrating lymphocytes (TILs) and their related cytokines, TLRs, innate immune cells, and others (Pagliari et al., 2018).TILs then produce pro inflammatory mediators that induce STAT3 and NF-kB pathways, which act as tumorigenic factors (Pagliari et al., 2018). This further increases cellular proliferation and suppresses apoptosis (Pagliari et al., 2018). Leaky gut is a potential possible mechanism for the development of PDAC. Fecal microbial transplant to overcome this immunosuppression might hold a future promise (Pitt et al., 2016).

#### Microbiome Role in Obesity

Disturbance in the gut microbiota is associated with metabolic syndrome (Mazidi et al., 2016; Saklayen, 2018). Studies show that the gut microbial compositions in genetically obese mice are different compared to those lean and wild-type under the same polysaccharide-rich diet (Tseng and Chun-Ying, 2019). Obese mice show a reduced abundance of *Bacteroidetes* and enrichment of *Firmicutes*. A fecal transplant from obese mice to germ-free mice resulted in a significant increase in the total body fat (Turnbaugh et al., 2006). Obese individuals show higher concentrations of lipopolysaccharide (LPS) leaking from the intestinal mucosa (Lau et al., 2021; Zawada et al., 2021). LPS stimulates the secretion of the satiety hormone (the pancreatic peptide hormone, PYY), which slows down the motility of the digestive tract and thus affects food absorption (Lau et al., 2021; Zawada et al., 2021). Other studies show that obese individuals had a significantly high abundance of *Firmicutes/Bacteroidetes ratio* (Tseng and Chun-Ying, 2019; Lau et al., 2021; Palmas et al., 2021). Members of *Firmicutes* such as *Ruminococcus gnavus* increase the production of SCFAs increasing intestinal energy harvesting and hepatic lipogenesis after carbohydrate-rich diets (Lau et al., 2021; Palmas et al., 2021). Some probiotics such as *Bifidobacterium animalis* ssp. *lactis* and *Lactobacillus gasseri* show promise in decreasing inflammation and intestinal leakage (Tseng and Chun-Ying, 2019; Lau et al., 2021; Schütz et al., 2021).

#### Microbiome Role in Coronary Artery Disease

Dysbiosis in the gut microbiome is associated with elevated blood pressure and consequently hypertension (Xu and Yang, 2020; Lau et al., 2021; Wang et al., 2021a). Hypertensive gut microbiomes had an increased abundance of opportunistic pathogens such as *Klebsiella* spp., *Streptococcus* spp., and *Parabacteroides merdae* (Yan et al., 2017). SCFAs producers such as *Roseburia* spp. and *F. prausnitzii* are lower in hypertensive patients (Yan et al., 2017). Previous studies showed a higher abundance of *Klebsiella*, *Clostridium*, and *Streptococcus* in patients with hypertension, which are known for their role as choline degraders (Hakenbeck et al., 2009; Craciun and Balskus, 2012; Kalnins et al., 2015). These patients also show overexpression of the choline utilization gene, *cutC* gene. The results suggest that choline intake and TMAO production might serve as diagnostic biomarkers for hypertension pathogenesis (Yan et al., 2017). Patients with coronary artery disease (CAD) showed an increase in *Lactobacillales*, and a decrease in *Bacteroidetes* (Yamashita et al., 2016), and a reduction in the abundance of *Roseburia intestinalis* and *F. prausnitzii* which are SCFAs producers (Jie et al., 2017; Xu and Yang, 2020). Reduction in secreted SCFAs leads to higher inflammation in CAD. In addition, the elevated gut permeability leads to an increased level of leaked LPS, which is associated with chronic inflammation in patients with post-myocardial infarction (Chambers et al., 2018; Zhou et al., 2018).

#### Microbiome Associations With Respiratory Diseases

Understanding the gut-lung axes is crucial to tackling lung disorders such as inflammatory and infectious diseases. The gut microbiome modulates the disease susceptibility of the lung and the lung microbiome alters the proinflammatory function of the gut microbiome (van der Lelie and Taghavi, 2020; de Oliveira et al., 2021; Lau et al., 2021). Since the start of the COVID-19 pandemic, microbiome scientists race to understand the role of the microbiome in disease development and diagnosis. Gut dysbiosis is associated with the translocation of bacteria into the blood during COVID-19. This potentially causes a secondary infection that might be life-threatening. The researchers observed an abundance of opportunistic pathogenic bacteria in hospitalized COVID-19 including antimicrobial-resistant species (Lau et al., 2021; Venzon et al., 2021). Leakage of bacteria into the bloodstream following dysbiosis is critical to control and can drive detrimental effects. COVID infection is associated with diarrhea in 40% of the patients, which in turn changes the GIT microbiome and induces inflammatory cytokines. *Ruminococcus gnavus* is positively correlated with inflammatory biomarkers (Villapol, 2020). Some gut microbiota is more enriched in COVID patients such as *R. gnavus*, *Ruminococcus torques*, and *Bacteroides dorei* (Lau et al., 2021; Yeoh et al., 2021). In-depth shotgun metagenomic analysis on fecal samples of COVID-19 patients revealed that *Coprobacillus*, *Clostridium ramosum*, and *Clostridium hathewayi* correlate with the severity of the COVID infection (Villapol, 2020). Dysbiosis in the gut microbiota is linked to several other lung disorders including pulmonary fibrosis, asthma, and cystic fibrosis. Pulmonary fibrosis patients show a higher abundance of some gut microbes including *Alloprevotella*, *Dubosiella*, *Helicobacter*, *OIsenella*, *Parasutterella*, *Rikenella*, and *Rikenllaceae*. This microbial enrichment is associated with an elevated level of some diseases biomarkers including trigonelline, betaine, cytosine, thymidine, glycerophosphocholine, taurocholate, adenine, deoxyadenosine, and deoxycytidine (Gong et al., 2021; Lau et al., 2021). Asthmatic kids show a significant reduction in *Bifidobacteria and an increase in Clostridia* (Anand and Mande, 2018). Some studies suggest that the lung microbiome and gut microbiome are linked. For example, an infection with the influenza virus in the respiratory tract of mice models increases the proportion of *Enterobacteriaceae* and reduces *Lactobacilli* and *Lactococci* in the gut microbiome (Looft and Allen, 2012; Enaud et al., 2020). The colonization of the gut by species of *C. difficile* at the age of 1 month is associated with asthma and wheezing at the age of 6–7 (van Nimwegen et al., 2011). A pioneering study revealed a significant alveolar microbiome signature associated with several lung diseases including interstitial lung diseases, chronic obstructive pulmonary diseases, and sarcoidosis (Gupta et al., 2021; Lau et al., 2021). Interestingly, the authors show a correlation between disease-specific microbiomes and lung normal flora (Gupta et al., 2021; Lau et al., 2021). Disease-specific microbiome is a promising target toward developing microbiome-based diagnostic biomarkers (Gupta et al., 2021; Lau et al., 2021). Further research is needed to understand the mechanisms of action of this association, which will be significant in developing new microbiome therapeutics and diagnostic biomarkers.

### Microbiome Role in Psychiatric, Behavioral, and Emotional Disorders

The gut-brain axis is one of the hot topics in microbiome research (Salagre et al., 2017; Jang et al., 2020; Mayer et al., 2022). Microbiome associations are evident in many psychiatric and neurological disorders including anxiety, and depression.

#### Microbiome Association With Anxiety Disorder

The gut microbiome affects brain function early from the womb life. A study shows that maternal microbial alterations impact offspring brain maturation in mice (Buffington et al., 2016). Mice offspring that were fed high-fat diets were observed to have a 9-fold reduction in *Lactobacillus reuteri* and oxytocin-producing cells (Buffington et al., 2016). While mice offspring exposed to maternal stress show a low abundance of *Bifidobacteria* and *Lactobacilli*. This microbial shift is associated with a decrease in cognitive functioning and an increase in anxiety disorder (Golubeva et al., 2015). This study has further experimented in GF mice. GF mice showed anxious behavior and increased serotonin levels in the hippocampus compared to mice with normal flora. A genome-wide association study revealed 141 host genes and microbial taxa that are implicated in anxiety and depression in mice (Jin et al., 2021; Lau et al., 2021). The study shows that some of these host genes control the structure of the gut microbiome by modulating specific taxa and hence influence anxiety indirectly. The study suggests an interesting approach to consider both host and microbiome genes when assessing or treating anxiety (Jin et al., 2021; Lau et al., 2021). Microbiome taxa that are enriched in anxiety disorder include Ruminococcaceae, Clostridiaceae, and Clostridiales. While Bacteroidales and Bacteroidaceae showed lower abundance (Jin et al., 2021; Lau et al., 2021).

#### Microbiome Association With Depression

Research shows an association between depression and some microbial taxa such as *Eggerthella*, *Subdoligranulum*, *Coprococcus*, and *Ruminococcaceae* (Lau et al., 2021; Radjabzadeh et al., 2021). Some microbial species are involved in metabolic pathways linked to neurological functions such as glutamate, butyrate, serotonin, and gamma aminobutyric acid neurotransmitters (Lau et al., 2021; Radjabzadeh et al., 2021). These microbes include *Lachnoclostridium*, *Sellimonas*, *Ruminococcaceae*, *Lachnospiraceae*, *Hungatella*, *Ruminococcus gavreauii*, and *Eubacterium ventriosum* (Lau et al., 2021; Radjabzadeh et al., 2021). In another study, a microbiome analysis revealed that patients with major depressive disorders had an increased abundance of the genus *Bacteroides* and decreased abundance of *Blautia* and *Eubacterium* (Lau et al., 2021; Yang et al., 2021). *Bacteroides* species are also known for their regulation of metabolic pathways and the immune system (Maier et al., 2015). *Bacteroides* modulate cytokines production and increase inflammation (Schiepers et al., 2005). In contrast, *Balutia* species mediates beneficial anti-inflammatory effects (Bajaj et al., 2012). Another study showed that *Clostridiales* and *Desulfovibrionaceae* are enriched in depressed patients, while *Bacteroidaceae* show low abundance (Lau et al., 2021; Rhee et al., 2021). However, we still lack information about the directionality of this microbiome association and the underlying mechanism.

#### Microbiome Role in Sleep Disorder

The gut microbiome composition is linked to sleep behavior (Maki et al., 2020). Disrupted sleep is linked to dysregulation in the immune system which leads to abnormal increases in inflammatory responses (Irwin, 2019). Sleep dysfunction can promote inflammation *via* two processes. First is through the upregulation of proinflammatory cytokines such as IL-6, tumor necrosis factor α (TNFα), and CRP (Matenchuk et al., 2020). The second is through the change in microbiome composition and function. In mice with sleep fragmentation (SF), the relative abundance of *Firmicutes* and the *Firmicutes/Bacteroidetes* ratio is reduced accompanying a decrease in butyrate-producing bacteria (Maki et al., 2020). Other studies found differences in *Bacteroidetes*, *Firmicutes*, *Lachnospiraceae*, or *Blautia* between short and normal sleepers (Anderson et al., 2017; Smith et al., 2019; Grosicki et al., 2020). Short sleepers showed a significantly lower abundance of *Sutterella* and a higher abundance of *Pseudomonas* (Agrawal et al., 2021; Lau et al., 2021). In a similar study, sleep-disturbed mice showed a shift in both the microbiome and metabolome. *Firmicutes/Bacteroidetes* ratio increased in the sleep-disrupted group along with a decrease in the *Lactobacillus*, *Actinobacteria*, and *Bifidobacterium* in comparison to control mice (Bowers et al., 2020). Studies investigated the role of probiotics in improving sleep with some promising preliminary results. For example, *L. gasseri* CP2305 shows some potential to improve the quality of sleep (Nishida et al., 2017; Matenchuk et al., 2020).

#### Microbiome Role in Fear Response

Fear is an emotional and behavioral response induced by a threat. Fear is considered an evolutionarily conserved mechanism that promotes survival (Carlson et al., 2021; Lau et al., 2021). A recent study found that babies with less balanced gut microbiomes show an increase in fear behavior in comparison to those with more balanced microbiomes (Carlson et al., 2021; Lau et al., 2021). Gut microbiome communities dominated by *Bacteroides* at the first year of age are associated with less non-social fear, while the lower abundance of *Bacteroides* and increased abundance of *Veillonella*, *Dialister*, *Bifidobacterium*, and *Lactobacillus* were linked to increased fear behavior (Carlson et al., 2021; Lau et al., 2021). In addition, the researchers found an association between the microbiome and the medial prefrontal cortex volume and amygdala volume at the first year of age. Infants who had an increased fear activity also had larger amygdala volumes (Carlson et al., 2021; Lau et al., 2021). Cutting the vagus nerve did not have any effect on the ability to extinguish fear in mice (Chu et al., 2019). The immune cells are the same in germ-free mice and antibiotic-treated mice. The fear response was associated with a reduction in some microbial metabolites in the cerebrospinal fluid, blood serum, and feces of GF mice. These metabolites include phenyl sulfate, pyrocatechol sulfate, and indoxyl sulfate (Chu et al., 2019). These findings suggest that microbiome metabolites might play a role in influencing brain activity, which then alters how mice extinguish fear memory. Administration of *L. helveticus* NS8 probiotic shows anxiolytic and antidepressant effects in rats with a restored level of corticosterone (Liang et al., 2015). This indicates that *L. helveticus* NS8 probiotics have a role in regulating the hypothalamic-pituitary adrenocortical (HPA) activation axis (Liang et al., 2015).

#### Microbiome Role in Autism

Children with autism spectrum disorder (ASD) show differential gut microbiome composition (Wang et al., 2011; Coury et al., 2012; Chaidez et al., 2014; Kheirouri et al., 2016). The microbiome in ASD shows a decrease in the abundance of *Escherichia*, *Shigella*, *Veillonella*, *Akkermansia*, *Providencia*, *Dialister*, *Bifidobacterium*, *Streptococcus*, *Ruminococcaceae*, *Megasphaera*, *Eubacterium_coprostanol*, *Citrobacter*, *Ruminiclostridium*, and *Ruminiclostridium*, while *Eisenbergiella*, *Klebsiella*, *Faecalibacterium*, and *Blautia* are significantly increased (Lau et al., 2021; Ye et al., 2021). Similarly, another research reveals that patients with ASD exhibit a lower *Bacteroidetes/Firmicutes* ratio (Kang et al., 2013). This alteration in the gut microbiome of ASD patients might be of diagnostic value for early detection of the disease. Children with ASD and gastrointestinal symptoms show higher levels of gut immune inflammation and gut dysbiosis (Hughes et al., 2018; Liu et al., 2021). Probiotics can reduce gut inflammation decreasing gut leakage, and downregulate inflammatory cytokines (Ng et al., 2019). The use of probiotics containing *Lactobacillus* and *Bifidobacteria* strains has been shown to ameliorate Gastrointestinal symptoms (Zhu et al., 2020). Another study showed that administration of *L. reuteri* improves social behaviors in autism model mice through signaling mechanisms *via* a nerve from the gut to the brain (Poutahidis et al., 2013; Sgritta et al., 2019).

#### Microbiome Role in Alzheimer and Dementia

Neurodegenerative diseases are characterized by the formation of amyloid plaque due to the aggregation of misfolded proteins in the neurons. To study the link between gut microbes and protein aggregation, *Caenorhabditis elegans* is used as a model organism. A study demonstrates that colonization of *C. elegans* gut with bacterial pathogens disrupted the intestines, muscles, and neurons and increased protein aggregation (Lau et al., 2021; Walker et al., 2021). Further investigation revealed that co-colonization with butyrogenic bacteria inhibited the aggregation of protein in *C. elegans* indicating that enteric bacteria play a role in the pathogenesis of neurodegenerative diseases such as Alzheimer’s disease (Lau et al., 2021; Walker et al., 2021). Other studies show that SCFAs production is altered in patients with neurodegenerative disease (Lau et al., 2021; Tran and Mohajeri, 2021). These metabolites might induce changes in the brain suppressing inflammation and cytokines production (Zhu et al., 2020). Emerging research investigates the use of probiotics as a therapeutic tool to suppress symptoms and delay the progression of Alzheimer’s (Peterson, 2020). Early results show that *Lactobacillus*, *Bifidobacterium breve*, and *B. longum* administration improved memory and learning dysfunction in animal models and patients with Alzheimer’s (O’Sullivan et al., 2011; Savignac et al., 2013; Peterson, 2020; Zhu et al., 2020). Microbiome composition is different in patients with dementia disorders compared to healthy populations. A study shows that *Clostridiales*, *Lactobacillus*, and *Bacteroidales* are enriched in patients (He et al., 2018a). This altered microbial composition is associated with an elevated high level of SCFAs and choline, the latter is a biomarker for membrane dysfunction (He et al., 2018b). Another study shows a significant reduction in *Bacteroides* and enrichment of *Lactobacillales* and *Bifidobacterium* in dementia patients (Saji et al., 2019). A possible mechanism for this observed link between gut microbiome composition and brain function might be the leaky gut. The leakage of harmful bacteria into the bloodstream affects mental health through immune regulation and oxidative stress (Belizário and Faintuch, 2018). Administration of probiotics might restore function or mediate symptoms of neurodegenerative diseases. Some preliminary data show that probiotics can improve cognitive function improving learning and memory in animal models (Lau et al., 2021; Ruiz-Gonzalez et al., 2021). However, future studies are necessary to understand the underlying mechanisms before proceeding to clinical trials in humans.

## Understanding the Microbiome Function as Mediated by Secreted Molecules

**Figure 3:**
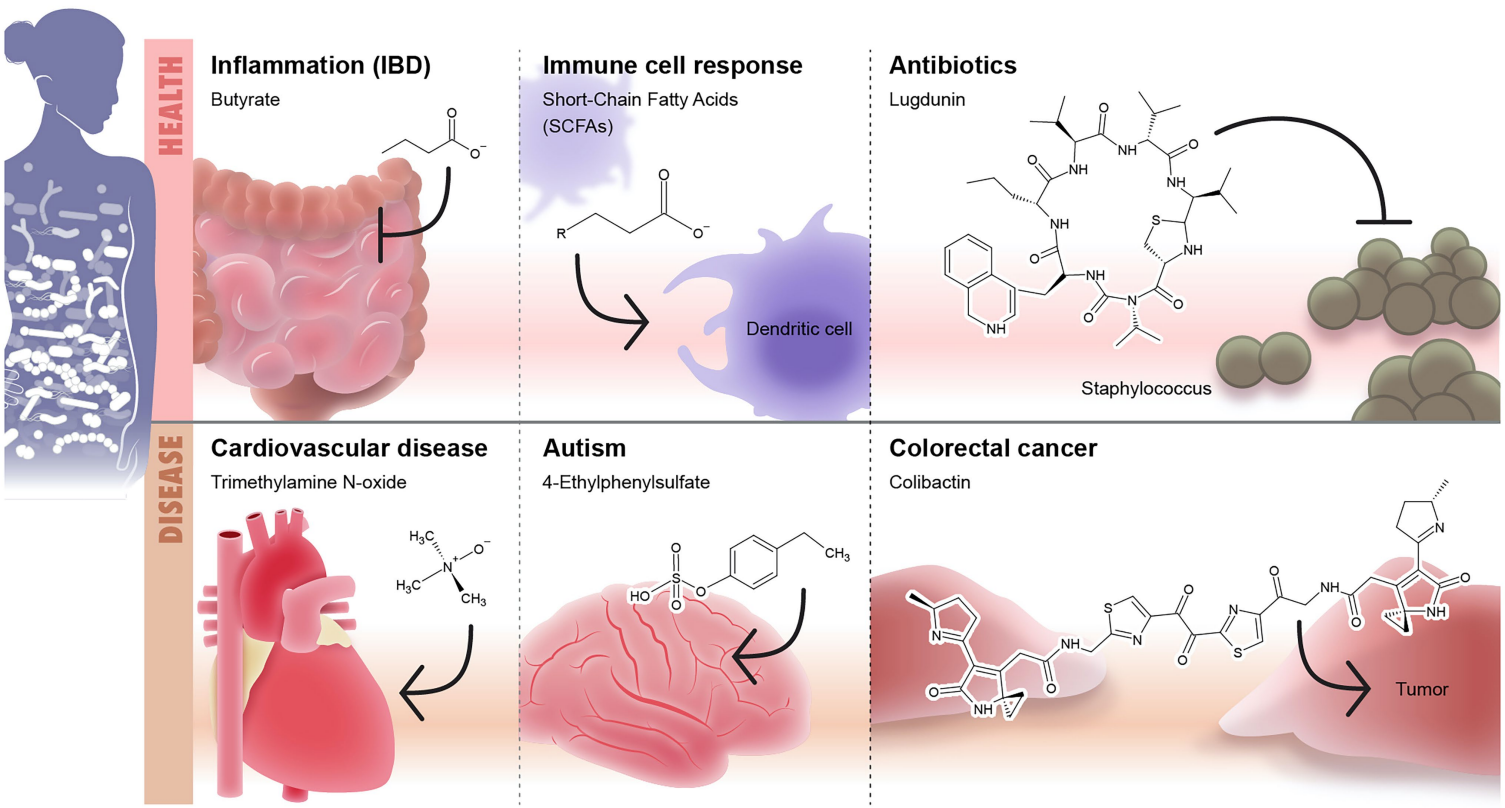
Microbiome-secreted molecules and their effect on human health and diseases. The first panel of the Illustration shows some examples of well-defined secreted molecules that affects human health including (1) short-chain fatty acids (SCFAs) such as butyrate which play anti-inflammatory role and modulate the intestinal immunity and (2) lugdunin as an example to microbiome-based antibiotic produced by nose microbiome and target *Staphyloccous*. The second panel shows examples of microbiome-based metabolites that are associated with onset or development of diseases including: (1) trimethylamine N-oxide (TMAO)/cardiovascular diseases, (2) 4-ethylphenylsulphate/autism, and (3) colibactin/colorectal cancer.

Commensal microbes are known to metabolize indigestible materials, defend against colonization of pathogens, and stimulate the immune system (Martín et al., 2013). Little is known about microbiome secreted molecules that drive the microbiome function and disease associations. Microbiome chemistry is what mediates microbe-host interaction and further disease susceptibility. Here, we discuss and highlight some microbiome known chemistry (Figure 3) and the future promise to develop microbiome-based therapeutics.

### Trimethylamine N-Oxide

The gut microbiome utilizes dietary precursors such as choline, phosphatidylcholine, and L-carnitine to produce trimethylamine N-oxide (TMAO), which is a mediator between intestinal dysbiosis and vascular pathology (Lau et al., 2021; Sikora et al., 2021). TMAO mediates effective attraction between lipid membranes by partitioning unevenly between bulk and lipid domains (Sukenik et al., 2017). If there is damage in the intestinal barrier such in chronic psoriasis patients (Sikora et al., 2018, 2019a,b), TMA leaks into the systemic circulation and get transformed by hepatic enzymes into TMAO, which triggers a strong inflammatory response through activation of mitogen-activated protein kinase (MAPK) and subsequent cardiovascular diseases. TAMO producers include *Clostridiaceae* and *Peptostreptococcaceae* (Yamashita et al., 2016). Other research suggests that TMAO precursors drive toxic effects on the cardiovascular system (Jaworska et al., 2019). TMAO-induced inflammation could be suppressed by antibiotics (Janeiro et al., 2018). Zhu et al. (2020) showed that the formation of TMAO in microbes could be inhibited by 3,3-dimethyl-1-butanol (DMB). TMAO is further involved in the development of cancers and neurological disorders (Janeiro et al., 2018; Gatarek and Kaluzna-Czaplinska, 2021). TMAO is also linked to atherosclerosis and inhibition of hepatic bile acid synthesis; although, the underlying mechanism is lacking (Stubbs et al., 2016; Gatarek and Kaluzna-Czaplinska, 2021).

### Colibactin

Colibactin is a microbial product associated with DNA damage leading to colorectal cancer (CRC; Putze et al., 2009; Arthur et al., 2012; Dziubańska-Kusibab et al., 2020). Colibactin is biosynthesized by deacetylation of the inactive precursor, precolibactin, by the peptidase enzyme, CIbP (Dziubańska-Kusibab et al., 2020). Colibactin alkylates DNA through its unique chemistry that includes a cyclopropane ring conjugated to an α,β-unsaturated imine. This creates adenine–colibactin adducts and then DNA crosslinks (Wilson et al., 2019; Xue et al., 2019; Dziubańska-Kusibab et al., 2020). Research predicts that these lesions in the DNA lead to mutations that promote CRC development (Dziubańska-Kusibab et al., 2020). In another study, colibactin showed an association with bowel cancer (Pleguezuelos-Manzano et al., 2020). The human cells treated with colibactin had twice the rate of DNA damage compared to the control group with some mutations that were only found in the colibactin-exposed cells (Pleguezuelos-Manzano et al., 2020). There were two mutation types: small indel signatures called ID-*pks* and a single base substitution signature called SBS-*pks* (Pleguezuelos-Manzano et al., 2020). This is evidence of the significant role of colibactin in GIT malignancy.

### Ethylphenyl Sulfate and Phenylalanine

The microbial metabolite 4-Ethylphenyl sulfate (4EPS) plays a role in the development of autism-related behavioral disorders in mice (Hsiao et al., 2013). Several species of *Clostridium* can produce the precursors of 4EPS which is 4-ethylphenol (Nicholson et al., 2012). Mice that are treated with 4EPS exhibited anxiety-like behavior suggesting that elevated levels of 4EPS cause ASD-related behaviors; thus reinforcing the notion that the connection between the gut and the brain might be associated with autism (Hsiao et al., 2013). In addition, researchers found that the blood of mice with autism symptoms had levels of 4EPS that were about 46 times higher than that of the control group (Hsiao et al., 2013). In a similar study, 4-EPS induced ASD-like behaviors (Zhu et al., 2020). A lower abundance of *Bacteroides* is linked to Autism (Hsiao et al., 2013). Introducing *Bacteroides fragilis* to mice with autism-like symptoms improved the symptoms (Hsiao et al., 2013) demonstrating that the probiotic treatment with *B. fragilis* might be helpful as a therapeutic intervention for autism. Phenylketonuria (PKU) is associated with autism development (Khemir et al., 2016). PKU is a metabolic disease in which the body cannot break down phenylalanine (Xiong et al., 2015; Blasco et al., 2017; Glinton and Elsea, 2019). An elevated level of phenylalanine and phenylpyruvate causes brain damage with a significant reduction in serotonin and dopamine (Glinton and Elsea, 2019), which might contribute to the development of autism if left untreated (Thompson et al., 1993; de Groot et al., 2010).

### Short-Chain Fatty Acids

Gut commensals produce SCFAs that inhibit pro-inflammatory responses mediated by intestinal macrophages (Chang et al., 2014). SCFAs production is altered in patients suffering from IBD (Zhuang et al., 2019). Butyrate is an example of SCFAs produced mainly by *Firmicutes*. Butyrate modulates the inflammatory immune response of intestinal macrophages. SCFAs modulate inflammation through multiple pathways include: (1) inhibiting adenylate cyclase which reduces the secondary messenger, cAMP (Houslay and Milligan, 1997; He et al., 2020), (2) activation of MAPK leading to an increase in Ca^2+^ concentration (Kimura et al., 2014; He et al., 2020), (3) stimulation of the release of anti-inflammatory interleukin 10 (IL-10) from the regulatory T cells, Tregs (Smith et al., 2013; He et al., 2020), (4) suppression of the expression of IL-6, IL-1β, and TNFα (Nakajima et al., 2017; Pirozzi et al., 2018; Mizuta et al., 2019; He et al., 2020), and (5) inhibiting histone deacetylase and downregulate lipopolysaccharide-induced pro-inflammatory mediators such as nitric oxide, IL-6, and IL-12 (Chang et al., 2014). Recent research shows that SCFAs bind to immune cell receptors in the respiratory tract and enhance lung antiviral response during infection with COVID-19 (de Oliveira et al., 2021; Lau et al., 2021). Beyond GIT, SCFAs are associated with changes in circulating immune cells and biomarkers, which are implicated in the development of multiple sclerosis (MS; Lau et al., 2021; Trend et al., 2021).

### Antimicrobial Compounds

A common trait for any polymicrobial ecosystem is the production of antibiotics as an ecological fitness strategy to compete, survive, and thrive. The human microbiota is no different, dozens of antimicrobial compounds have been reported from the microbiome. Some examples of recently discovered antibiotics from the microbiome include Lugdunin, Lactocillin, cereulide, zwittermicin, tilivalline, and others (Table 3).

**Table 3:** Classes of antimicrobial compounds and their activity spectrum.

Group	Name of compound	Producing species	Target pathogens	References
Microcins	Microcin L	*Escherichia coli* LR05	*Shigella sonnei* *E. coli* *Pseudomonas aeruginosa*	Gaillard-Gendron et al., 2000
	Microcin M	*E. coli Nissile* 1917	*E. coli* and *Salmonella*	Patzer et al., 2003
	Microcin V	*E. coli*	*E. coli*	Cascales et al., 2007
	Microcin H47	*E.coli* H47	*E.coli*, *Salmonella*, *Enterobacter*, *Shigella*, *Klebsiella*, and *Proteus* spp.	Laviña et al., 1990
Lasso peptide	Microcin J25	*E. coli* AY25	*E. coli*, *Salmonella* sp., and *Shigella flexneri*	Salomón and Farías, 1992
Sactibiotics	Thuricin SD	*Bacillus thuringiensis* DP C 6431	*Clostridium difficile*, *Bacillus cereus*, *Bifidobacterium firmus*, and *Listeria monocytogenes*	Rea et al., 2010
IIa peptides	Bac43	*Enterococcus faecium* VRE82	*Enterococcus faecalis*, *E. faecium*, *Enterococcus hirae*, *Enteroccus durans*, and *L. monocytogenes*	Todokoro et al., 2006
	Bacteriocin 31	*E. faecalis* YI717	*E. hirae* 9790, *E. faecium*, and *L. monocytogenes*	Tomita et al., 1996
IIb peptides	ABP-118	*Lactobacillus salivarius* UCC-118	*L. monocytogenes*	Flynn et al., 2002
	Lactacin F	*Lactobacillus johansonii*	*Lactobacillus* sp., *E. faecalis*	Abee et al., 1994
IIc peptides	Gassericin A	*Lactobacillus gasseri* LA39	*Bacillus*, *Clostridium*, *Lactobacillus* spp., *Lactococcus lactis*, *Leuconostoc mesenteroides*, *Listeria* spp., *PEdiococcus cerevisiae*, *Staphylococcus aureus*, and *Streptococcus agalactiae*	Kawai et al., 1998
	Reutericin 6	*Lactobacillus reuteri* LA 6	*Lactobacillus acidophilus*, *Lactobacillus delbrueckii* subsp. *Bulgaricus*, and *L. delbrueckii* subsp. *lactis*	Toba et al., 1991
IId peptides	Microcin S	*E. coli* G3/10	*E. coli* G3/10	Beimfohr, 2016
	Rhamnosin A	*Lactobacillus rhamnosus* strain 68	*Micrococcus lysodeikticus* ATCC 4698	Dimitrijević et al., 2009
Bacteriolysin	Colicins	*E. coli*	*Enterobacteria*	Salomón and Farías, 1992
Non-lytic bacteriocins	Bacteriocin helveticin J	*L. acidophilus* NCFM	*NA*	Bull et al., 2013

Lugdunin is a thiazolidine-containing cyclic peptide produced by *Staphylococcus lugdunensis*, a commensal of the human nose (Konai et al., 2020). Lugdunin is active against both methicillin-resistant *S. aureus* and vancomycin-resistant enterococci (Konai et al., 2020). Lugdunin also has an immunomodulatory activity (Bitschar et al., 2019). Pretreatment of primary human keratinocytes or mouse skin with the antimicrobial lugdunin resulted in a significant reduction of *S. aureus* colonization (Bitschar et al., 2019). Lugdunin increases the expression and release of LL-37 and CXCL8/MIP-2 in human keratinocytes and mouse skin resulting in the release of monocytes and neutrophils (Bitschar et al., 2019). Lactocillin is another thiopeptide antibiotic produced by *L. gasseri*, a commensal of the vaginal microbiome (Vásquez et al., 2002). Lactocillin is active against *S. aureus*, *Enterococcus faecalis*, *Gardnerella vaginalis*, and *Corynebacterium aurimucosum* (Donia et al., 2014).

## Conclusion and Future Directions

Much interest exists in the potential of the evolved functions of the microbiome. A pioneering study aimed to computationally predict the function of microbes on earth estimates the presence of 35.5 million functions in bacteria of which only 0.02% are known (Starke et al., 2020). Despite the exploding body of research on the microbiome, our knowledge of its function and especially how it mediates health and diseases is still preliminary. The microbiome function is dependent on its structure and diversity, which is highly unique among individuals as shaped by multifactor. More dive into the individual’s unique microbiome might be a path to a personalized medicine approach. The drastic change in the microbiome of immigrants and the associated health consequences clearly demonstrates the structure and function dependency. Our knowledge of the change in microbiome composition with the onset or progression of diseases is based on association studies with a little dig into the mechanistic underpinning. The main concern in these studies is the lack of directionality of the microbiome disease relationship and the presence of other confounding factors. Recent research suggests that microbiome change in autism might be due to the picky eating habits restricting diet which in turn changes the microbiome composition (Lau et al., 2021; Yap et al., 2021). Another concern is that most association studies examine the change in the dominant microbial taxa, while recent studies show that rare microbes are the drivers of diseases. The use of microbiomes as probiotics or fecal transplant shows promise but with many challenges. Safety concerns are among the main challenges. One of the most studied and commercially available probiotic strains is *Escherichia coli* Nissle 1917 was found later to encode colibactin biosynthetic gene cluster implicated in CRC (Olier et al., 2012; Dziubańska-Kusibab et al., 2020). The second challenge is the efficacy and wide variation in response between individuals. In addition, introducing new microbes to an already established microbiome community comes with unpredicted outcomes include clearance from the body and failure to survive. Introducing the entire microbiome as in fecal transplant is another approach showing some efficacy especially in controlling recurrent *C. difficile* infection but with a complex and unpredictable outcome (Kazemian et al., 2020). The undesirable outcomes include the transfer of antibiotic resistance microbes (DeFilipp et al., 2019) and weight gain (Alang and Kelly, 2015). Recently the FDA issued a warning against the use of fecal transplant after two patients retracted antibiotic-resistant infections following administration of fecal transplant. A pioneering approach is designing of synthetic microbiome community with reproducible and controlled structure using an informatics approach (Clark et al., 2021; Lau et al., 2021). A synthetic microbiome community is drafted to supply a specific missing function such as butyrate synthesis for the treatment of inflammation (Clark et al., 2021; Lau et al., 2021). Personalized medicine based on microbiome signature of each individual is a direction that we should consider moving forward (Yadav et al., 2022). Microbiome signature could be used to assess diseases severity and prognosis, predict drug resistance or response rate, or even more importantly in diseases prevention (Behrouzi et al., 2019; Yadav et al., 2022). Certainly, the research on microbiome is exploding and the future of microbiome use in translation medicine is blooming.

## Author Contributions

FC and SH reviewed the literature, collected the data, contributed to writing, and developed the tables. WM reviewed the literature, collected the data, designed and developed the figures, wrote, and edited the manuscript. All authors contributed to the article and approved the submitted version.

## Funding

This work is supported by a startup fund from Whitman College and funding from Murdock foundation.

## Conflict of Interest

The authors declare that the research was conducted in the absence of any commercial or financial relationships that could be construed as a potential conflict of interest.

## Publisher’s Note

All claims expressed in this article are solely those of the authors and do not necessarily represent those of their affiliated organizations, or those of the publisher, the editors and the reviewers. Any product that may be evaluated in this article, or claim that may be made by its manufacturer, is not guaranteed or endorsed by the publisher.
